# Characterization of immune microenvironment infiltration and m^6^A regulator-mediated RNA methylation modification patterns in osteoarthritis

**DOI:** 10.3389/fimmu.2022.1018701

**Published:** 2022-11-23

**Authors:** Yulong Ouyang, Yuanqing Tu, Shuilin Chen, Huan Min, Zhexu Wen, Guihao Zheng, Ting Wan, Hao Fan, Wenzhao Yang, Guicai Sun

**Affiliations:** ^1^ Nanchang University, Nanchang, Jiangxi, China; ^2^ Jiangxi Provincial People’s Hospital, Nanchang, Jiangxi, China; ^3^ The Fourth Affiliated Hospital of Nanchang University, Nanchang, Jiangxi, China; ^4^ Shangrao People’s Hospital, Shangrao, Jiangxi, China; ^5^ The First Affiliated Hospital of Nanchang University, Nanchang, Jiangxi, China

**Keywords:** m^6^A regulator, immune microenvironment, osteoarthritis, RNA methylation, immunocytes

## Abstract

**Background:**

Few studies have been reported the potential role of N6-methyladenosine (m^6^A) modification in osteoarthritis (OA). We investigated the patterns of m^6^A modification in the immune microenvironment of OA.

**Methods:**

We evaluated the m^6^A modification patterns based on 22 m^6^A regulators in 139 OA samples and systematically associated these modification patterns with immune cell infiltration characteristics. The function of m^6^A phenotype-related differentially expressed genes (DEGs) was investigated using gene enrichment analysis. An m^6^A score model was constructed using principal component analysis (PCA), and an OA prediction model was established based on the key m^6^A regulators. We used real-time PCR analysis to detect the changes of gene expression in the cell model of OA.

**Results:**

Healthy and OA samples showed significant differences in the expression of m^6^A regulators. Nine key m^6^A regulators, two m^6^A modification patterns, m^6^A-related genes and two gene clusters were identified. Some m^6^A regulators had a strong correlation with each other. Gene clusters and m^6^A clusters have high similarity, and cluster A corresponds to a high m^6^A score. Immunocytes infiltration differed significantly between the two clusters, with the m^6^A cluster B and gene cluster B having more types of infiltrating immunocytes than cluster A. The predictive model can also predict the progression of OA through m^6^A regulators expression. The results of real-time PCR analysis showed that the gene expression in the cell model of OA is similar to that of the m^6^A cluster B.

**Conclusions:**

Our study reveals for the first time the potential regulatory mechanism of m^6^A modification in the immune microenvironment of OA. This study also sheds new light on the pathogenesis of OA.

## Introduction

Osteoarthritis (OA) is the most common joint disease worldwide, impacting 240 million individuals (1, 2). OA is a chronic joint disease, that is mainly characterized by pain, stiffness, joint deformity and limited joint activity (3). With the trend of the aging population and the obesity epidemic, this widespread disease and the resulting disability have a great impact on individuals and society (4). The progression of OA is driven by a series of factors, such as gene regulation, biochemical cascades, inflammation and cellular immunity (5, 6). However, the etiology and disease progression mechanisms of OA are still unclear, which limits the development of effective treatment (7).

Many studies have revealed the significance of epigenetics in disease progression (8, 9). Epigenetic modifications such as DNA methylation, RNA modification, histone modification and noncoding RNA modification have also been widely reported (10). Over 100 different types of RNA modifications have been discovered, including N1-methyladenosine (m^1^A), N6-methyladenosine (m^6^A), 5-methylcytosine (m^5^C), and N7-methylguanosine (m^7^G) (11). m^6^A RNA methylation occurs on approximately 20%-40% of all transcripts encoded by mammalian cells, and it is the most common type of dynamic and reversible mRNA modification (12, 13). Abnormal m^6^A methylation levels are strongly linked to the progression of cancer, musculoskeletal disorders and other diseases (10, 14). The level of m^6^A methylation is primarily determined by the role of the m^6^A regulator (15). Methyltransferases, demethylases, and binding proteins all play a role in m^6^A modification (16). The m^6^A methyltransferases (writers) include WTAP, METTL3, CBLL1, and RBM15B, while demethylases (erasers) consist of ALKBH5 and FTO. YTHDC1, YTHDF1, IGF2BP1, and other binding proteins (readers) can bind to m^6^A and mediate its regulatory function (12, 17). Previous studies have mostly concentrated on the role of m^6^A methylation regulators in tumor development and treatment (18–20). However, the number of studies of m^6^A regulators in nonneoplastic diseases is also increasing (21–23). There are only a few studies on the mechanism of m^6^A regulators and OA at present. Most of these studies clarify the mechanism of FTO and METTL3 in OA, but they are controversial. The mechanism of m^6^A regulators in OA is still unclear.

**Figure 1:**
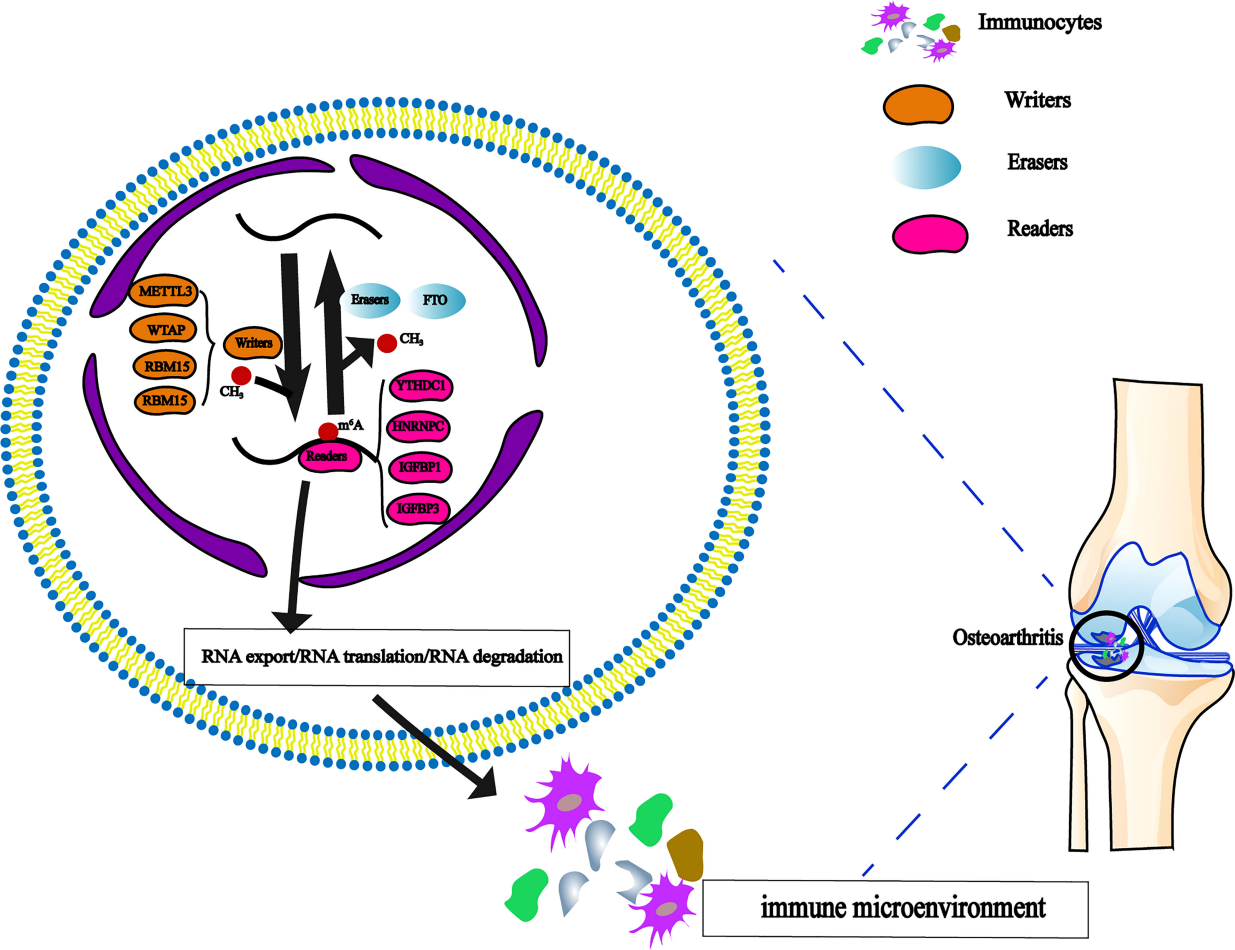
The dynamic regulation of m^6^A RNA methylation modification, which regulated by ‘writers’, ‘erasers’ and ‘readers’ in OA and their potential biological functions for RNA.

Recent research has revealed that m^6^A regulation can mediate some potential immune regulation mechanisms and has a significant impact on adaptive immunity (24, 25). More and more studies have focused on the effects of the immune microenvironment on diseases (26, 27). All types of immune cells are involved in cartilage injury and repair (28). However, the interaction of m^6^A methylation regulators with immune cells in OA is poorly understood (**Figure 1**).

This study aimed to systematically evaluate the mechanism of m^6^A regulators in OA. We analyzed the gene expression profile of OA by bioinformatics analysis. Subsequently, to further investigate the implication of m^6^A regulators on the immune microenvironment, we investigated the correlations among clustering subgroups, risk mode, and immune cell infiltration. These findings can provide a theoretical basis for the progress and treatment of OA.

## Materials and methods

### Datasets preprocess

The GSE48556 datasets were obtained from the Gene Expression Omnibus(GEO) database. Total mRNA was extracted from peripheral blood mononuclear cells and detected using the Illumina HumanHT-12 V3.0 expression beadchip (29). To preprocess the expression value, the “Normalize Between Arrays” function from the “limma” package was utilized.

### Analysis of m^6^A regulators between OA patients and healthy controls

We collated 22 recognized m^6^A methylation regulators from published literature (11, 12, 15, 30, 31). The following genes were screened: m^6^A readers (YTHDC1/2, YTHDF1/2/3, HNRNPC, FMR1, LRPPRC, IGFBP1/2/3, RBMX, ELAVL1, IGF2BP1), m^6^A writers (METTL3, WTAP, RBM15/15B, CBLL1, KIAA1429), and m^6^A erasers (FTO, ALKBH5). The Wilcoxon test was used to compare the expression of 22 m^6^A regulators in OA patients and healthy controls, and the differentially expressed m^6^A regulators were screened with P value < 0.05. The R package “Random Forest” and gene importance plots were used to show the score of differentially expressed m^6^A regulators. A nomogram was used to predict the possibility of OA in patients based on screened m^6^A regulators.

### Correlation of m^6^A RNA methylation regulators in OA patients

The correlation between m^6^A regulators in OA patients was investigated by the R package “corrplot” and Spearman’s correlation analysis. The R packages “ggMarginal” and “ggplot” are used to draw the correlation plot of significantly differentially expressed m^6^A regulators.

### m^6^A modification pattern identification

Unsupervised clustering analysis was used to detect diverse m^6^A clusters based on the 22 distinct m^6^A regulators’ expression. The number and feasibility of modification patterns were identified by the consensus clustering algorithm. To categorize OA patients into distinct subtypes, we utilized the “ConsensusClusterPlus” software (1,000 iterations and an 80% resampling rate). The m^6^A-expression pattern was assessed by principal component analysis (PCA). The m^6^A regulators’ differential expression in different m^6^A clusters is shown in the box plot and heatmap. We analyzed the difference in the levels of cytokines among m^6^A clusters.

### Immune cell infiltration analysis

We use the ssGSEA algorithm to calculate the enrichment score of immunocytes infiltration in each sample. The gene set of infiltrating immune cells was obtained from previous studies, which included activated B cells (ABCs), type 1/2/17 T helper (Th1/2/17) cells, dendritic cells (DCs), natural killer (NK) cells and other 23 human immune cell subtypes (32). The combined ssGSEA score was used to compare the levels of immunocytes infiltration in distinct m^6^A clusters. The determining criterion of a significant difference was P < 0.05. The correlation between major m^6^A regulators and immunocytes infiltration was determined by Spearman correlation analysis. According to the expression of m ^6^A regulators, which are strongly related to immune cell infiltration, OA patients were divided into two groups. Then, the difference in immunocytes infiltration levels in different subgroups was calculated (P value<0.05).

### Biological enrichment analysis for distinct m^6^A modification patterns

We identified the DEGs among distinct m^6^A subgroups and the overlapping genes were extracted (|log FC|>1, adjusted P value<0.05). GO functional analysis and KEGG pathway enrichment analysis were performed to analyze the DEGs *via* R packages (enrichplot, circlize, RColorBrewer, dplyr, ComplexHeatmap, and so on). A P value of 0.05 was used as the cutoff.

### Identification and analysis of gene clusters

We used the extracted overlapping genes and the clustering algorithm to determine cluster numbers and stability. We determined the OA gene cluster based on the extracted overlapping genes and unsupervised clustering analysis. We used a box plot diagram to show the m^6^A regulators as differentially expressed in different gene clusters. The degree of immunocytes infiltration and expression of interleukin-associated factors were compared between gene clusters. The method was consistent with the immune infiltration analysis of m^6^A clusters.

### Construction of m^6^A gene signature

The m^6^A clusters and gene clusters were identified by previous methods. Then, we constructed the m^6^A gene signature by PCA. As feature scores, principal components 1 and 2 were retrieved. The score was based only on the most significantly correlated genes, while untracked genes’ contributions to other set members were weighed. The calculation method for determining the m^6^A gene signature score was based on previous studies (m^6^Ascore =∑ (PC1_i_ + PC2_i_), i = the expression of m^6^A phenotypic related genes) (33, 34). The difference in the m^6^A score in m^6^A clusters and gene clusters was analyzed.

### Cell and cell culture

The human chondrosarcoma cell line SW135 (Pricella, China) was maintained in Dulbecco’s modified Eagle’s medium(DMEM)-high glucose (Gibco, United States) containing 10% Fetal Bovine Serum (FBS, Gibco, United States) and 1%penicillin/streptomycin. The chondrogenic ATDC5 cell line (Riken Cell Bank, Japan) was cultured in DMEM/F12 (Keygen, China) containing 10% FBS and 1%penicillin/streptomycin. Before the following experiments, all the cells were maintained under standard adherent conditions at 37°C under 5% CO_2_ and humidified atmosphere.

### Real-time PCR analysis

SW1353 cells and ATDC5 cells were treated with 10 ng/ml recombinant human IL-1β (Proteintech, Rosemont, IL) for 48 h (35). Total RNA was extracted from cells using RNAiso plus reagent (Takara, Japan) according to the manufacturer’s instructions. RNA (1 µg) was reverse-transcribed to complementary DNA using cDNA Synthesis Super Mix (Trans Gen Biotech, China) according to the protocol of the manufacturer. The primers used for amplification are listed in the table below (**Table 1**). Green qPCR Super Mix (Trans Gen Biotech, China) was used for real-time PCR using the CFX Connect Real-Time PCR Detection System (Biorad, United States). The thermal cycling conditions were 95°C for 30 s and 42 cycles at 95°C for 5 s and 60°C for 30 s.

**Table 1:** The sequences of primer pairs used in the study.

Gene	Forward	Reverse
H:GAPDH	5’-GGAAGCTTGTCATCAATGGAAATC-3’	5’-TGATGACCCTTTTGGCTCCC-3’
H:Mettl3	5’-CAGCACAGCTTCAGCAGTTCC-3’	5’-CGTGGAGATGGCAAGACAGA-3’
H:Wtap	5’-CCAAGAAGGTTCGATTGAGTGA-3’	5’-CAGACTCCTGCTGTTGTTGCTTT-3’
H:RBM15B	5’-CACAGCGTATCTGAGGTGGAG-3’	5’-GTTCTGGAACTTGAGGAAGGCATA-3’
H:Rbm15	5’-GTGAGCGGAGCAAGAAGTTAGG-3’	5’-CTATAACTATGCAAGCGGCTACTG-3’
H:YTHDC1	5’-GAGGGAATTTCATAACATGGGAC-3’	5’-ATGGTGCTGATAGTAAGGATGGTGT-3’
H:Fto	5’-GCCAGGTGCCAGTCACGAAT-3’	5’-TGTGAGGTCAAACGGCAGAG-3’
H:HNRNPC	5’-CGCTCCATGAACTCCCGTGT-3’	5’-GTTCTGTTACTGACCCGTACATCTC-3’
H:IGFBP1	5’-GCACGGAGATAACTGAGGAGGA-3’	5’-TCTTGTTGCAGTTTGGCAGGTA-3’
H:IGFBP3	5’-CTCAGAGCACAGATACCCAGAAC-3’	5’-AGGCTGCCCATACTTATCCAC-3’
M:GAPDH	5’-CCTCGTCCCGTAGACAAAATG-3’	5’-TGAGGTCAATGAAGGGGTCGT-3’
M:Mettl3	5’-AGGACTCTGGGCACTTGGATT-3’	5’-ATGGCAAGACGGATGGAAAC-3’
M:Wtap	5’-GCAAGATGACCAACGAAGAACC-3’	5’-TTAACTCATCCCGTGCCATAAC-3’
M:RBM15B	5’-CACAGTGTTTCTGAGGTGGAGC-3’	5’-GCCTTGCCGTAGCCTATCTTT-3’
M:Rbm15	5’-GTTCAAACGCTTCGGTGATGTA-3’	5’-CACAAAGGCTACCCGCTCAT-3’
M:YTHDC1	5’-CCGATACCAGGAAGTGGACAGAC-3’	5’-TGGTGCTGGTAGTAAGGATGGTG-3’
M:Fto	5’-TGAGGTGGAGTTTGAGTGGCTG-3’	5’-AGAATCTCACTCCTTTGTTCCACC-3’
M:HNRNPC	5’-ACGGTTCCTCATTTGACTTGG-3’	5’-AACACGCTGACGTTTGGAAGG-3’
M:IGFBP1	5’-GCCACGAGCACCTTGTTCAG-3’	5’-GCAGGGCTCCTTCCATTTCTT-3’
M:IGFBP3	5’-CTCAAAGCACAGACACCCAGAA-3’	5’-GGCGGCACTGCTTCTTCTTAT-3’

(H, Human; Used in the SW1353. M, Mouse; Used in the ATDC5).

### Statistical analysis

R 4.1.3 (https://www.rproject.org/), Perl 5.32.1 (https://www.perl.org) and R Bioconductor packages were used to analyze the data. Statistical analyses of real-time PCR were performed using GraphPad Prism software (version 9.0). Statistical significance was assessed using the student t-test. All experiments were performed independently at least three times. All the statistical P values were bilateral, with P value less than 0.05 considered statistically significant.

## Results

### Expression of m^6^A regulators in OA

**Figure 2:**
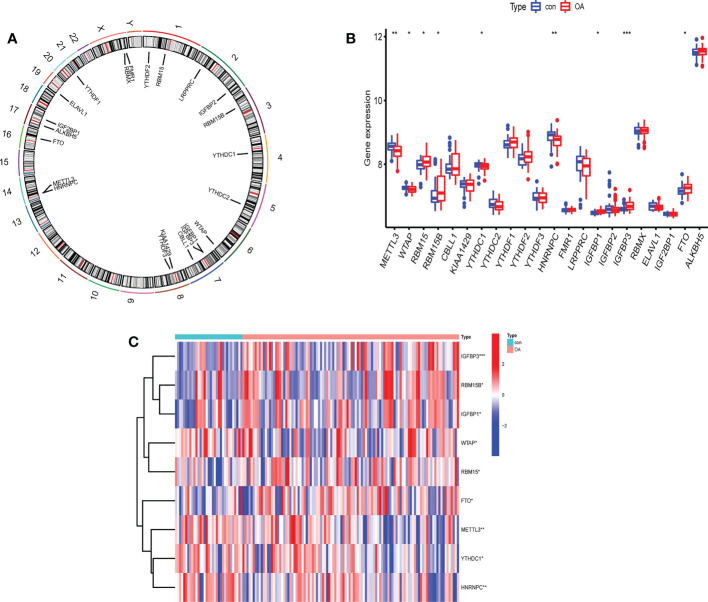
The expression levels of screened m^6^A regulators in healthy controls and OA patients. **(A)** The location of 22 RNA methylation m^6^A regulators on human chromosomes. **(B)** The expression levels of 22 m^6^A RNA methylation regulators between healthy controls and OA patients (*P < 0.05, **P < 0.01, ***P < 0.001). **(C)** Heatmap of expression levels of 9 key m^6^A RNA methylation regulators in healthy controls and OA patients (*P < 0.05, **P < 0.01, ***P < 0.001).

The profile expression data consisted of 139 samples, including 106 genetics osteoarthritis and progression (GARP) samples and 33 normal samples. The positions of 22 selected m^6^A regulators on the chromosomes are marked in **Figure 2A**. The expression levels of 22 m^6^A regulators in healthy patients and OA patients are shown in **Figure 2B**. There were significant differences in 9 m^6^A regulators (METTL3, WTAP, RBM15/15B, YTHDC1, HNRNPC, IGFBP1/3, and FTO) between normal and OA samples. In OA, four m^6^A regulators (YTHDC1, HNRNPC, METTL3, and WTAP) were downregulated and five m^6^A regulators (RBM15, RBM15B, IGFBP1, IGFBP3, and FTO) were upregulated (**Figure 2C**). IGFBP3 expression was dramatically higher in the peripheral blood of OA patients (P<0.001). METTL3 and HNRNPC levels in OA patients were considerably lower than those in healthy controls (P<0.01). Thus, these m^6^A regulators are critical in OA.

**Figure 3:**
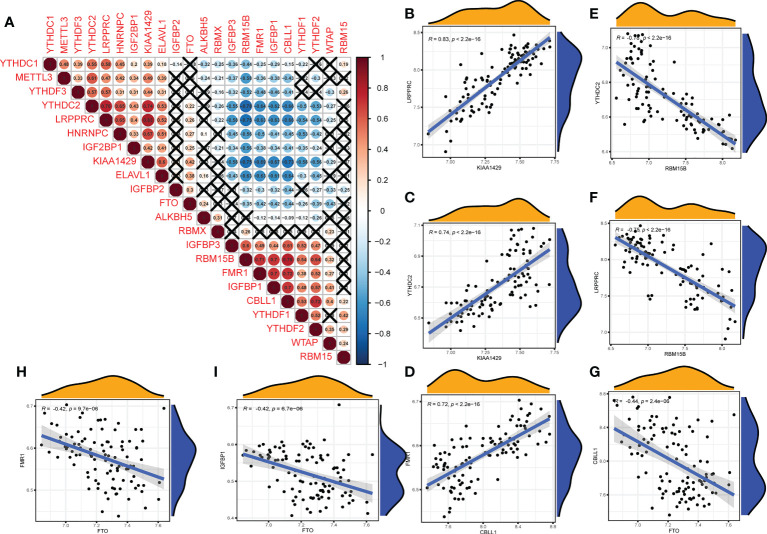
Correlation of m^6^A regulators in OA patients. **(A)** The relationship between m^6^A RNA methylation regulators in OA (A fork indicates P>0.05). **(B–F)** The correlation among writers and readers with a strong correlation coefficient in OA. **(G)** The correlation among erasers and writers in OA. **(H, I)** The correlation among erasers and readers in OA **(B–I)** Comparison between regulators with strong correlation coefficient).

We further analyzed the correlations among the 22 m^6^A regulators in the OA group (**Figure 3A**). Some m^6^A regulators had a strong correlation with each other (**Figures 3B–F**: writers and readers, **Figure 3G**: erasers and writers, **Figures 3H, I**: erasers and readers).

### m^6^A methylation modification patterns mediated by regulators

**Figure 4:**
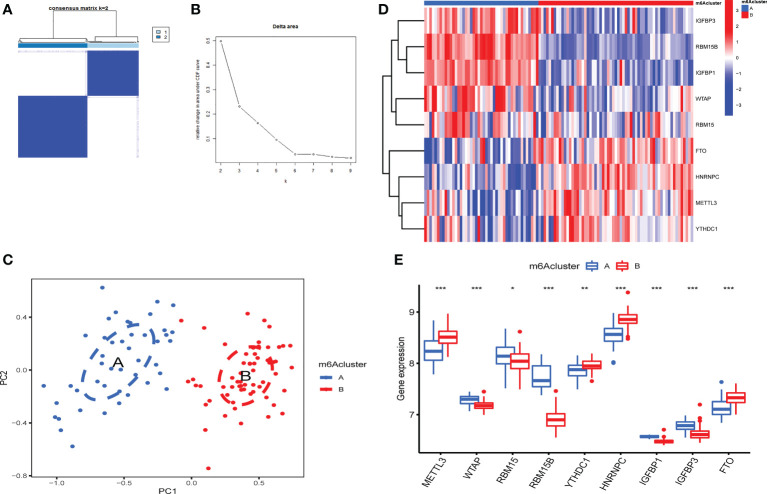
Identification of 2 distinct m^6^A modification patterns in OA. **(A)** Consensus clustering matrix (CCM) with K=2. **(B)** Change in the area under the cumulative distribution function (CDF) curve for k from 2 to 9. **(C)** PCA showed that two m^6^A clusters did not intersect (2 modification patterns in OA are an appropriate choice). **(D)** Heatmap of the expression of 9 key m^6^A RNA methylation regulators in 2 m^6^A clusters. **(E)** Box plot of the expression of 9 key m^6^A regulators in the 2 m^6^A modification patterns (*P < 0.05, **P < 0.01, ***P < 0.001).

According to m^6^A regulator expression, the clustering stability was analyzed with cluster numbers from 2 to 9 (**Figure 4A** and **Figure 1S**). The optimal k value was determined, and k = 2 was eventually selected as the optimal cutoff (**Figure 4B**). PCA revealed that OA patients could be classified into two m^6^A clusters that did not intersect. Thus, two is an appropriate cluster number (**Figure 4C**). There were 45 OA patients in cluster A and 61 in cluster B. IGFBP1, IGFBP3, RBM15B, RBM15 and WTAP were highly expressed in cluster A. FTO, HNRNPC, METTL3 and YTHDC1 were expressed at lower levels in cluster A than in cluster B (**Figures 4D, E**).

### Immune microenvironment features related to two m^6^A modification patterns

**Figure 5:**
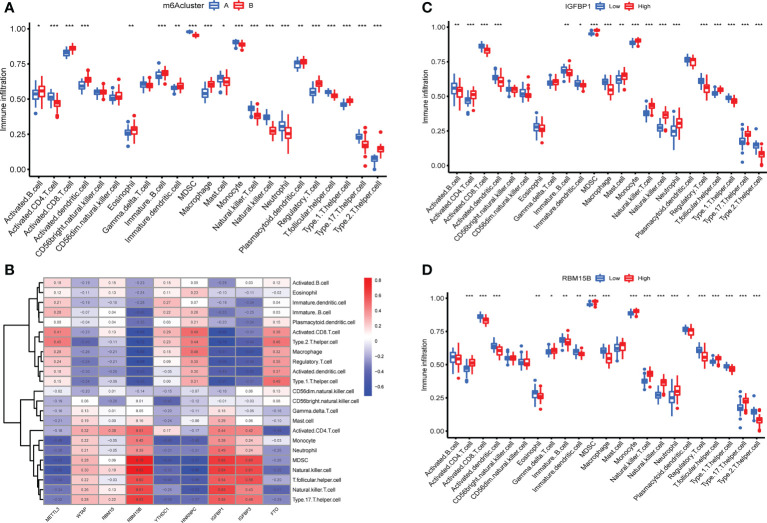
Immune microenvironment features in two m^6^A modification patterns. **(A)** Levels of infiltrating immunocytes in two m^6^A clusters in OA (*P < 0.05, **P < 0.01, ***P < 0.001). **(B)** Correlation between 9 key m^6^A regulators and immune cells. **(C)** The effect of high and low expression of IGFBP1 on immune infiltration (*P < 0.05, **P < 0.01, ***P < 0.001). **(D)** The effect of high and low RBM15B expression on immune infiltration ns, P>0.05, No statistical difference; (*P < 0.05, **P < 0.01, ***P < 0.001).

The expression of immune cells was examined to demonstrate the changes in immune microenvironment features in distinct m^6^A modification patterns. Between the two m^6^A clusters, there were substantial variations in virtually all immune cells observed (**Figure 5A**). Compared with m^6^A cluster A, the cluster B had a higher level of ABCs, activated CD8^+^ T cells, activated DCs, eosinophils, immature B cells, immature DCs, macrophages, plasmacytoid DCs, regulatory T cells, Th1 cells and Th2 cells. Activated CD4^+^ T cells, MDSCs, mast cells, monocytes, NK T cells, NK cells, neutrophils, T follicular helper cells and Th17 cells were enriched in cluster A. More immune cells are enriched into the cluster B.The correlation between the screened m^6^A regulators and immunocytes was investigated. The infiltration of immunocytes was substantially linked with the levels of IGFBP1 and RBM15B (**Figure 5B**). OA patients were separated into two groups based on the difference in the expression of IGFBP1 or RBM15B. Although 9 types of immunocytes were enriched in the 2 IGFBP subgroup, the high IGFBP expression group may have a higher level of immunocytes infiltration (**Figure 5C**). In addition, the high RBM15B expression group had fewer kinds of immune cells (**Figure 5D**).

### Biological characteristics of m^6^A modification patterns

**Figure 6:**
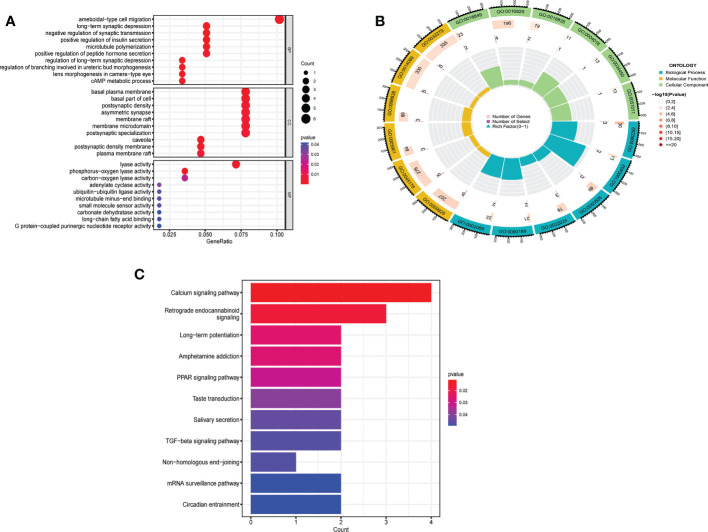
Biological characteristics related to m^6^A modification patterns. **(A)** GO analysis of m6A phenotype-related DEGs from three perspectives: biological process, cellular composition and molecular function. **(B)** The circle diagram of gene enrichment numbers for each GO item. **(C)** KEGG pathway enrichment analysis of m^6^A phenotype-related DEGs.

Between m^6^A clusters A and B, 303 genes (m^6^A phenotype-related genes) were differentially expressed. GO enrichment analysis was used for analysis of enriched biological processes, molecular functions, cellular components. These genes are mostly associated with ameboidal-type cell migration (biological process), basal plasma membrane (cell component), and lyase activity (molecular function) (**Figures 6A, B**). To investigate the relevant activities and pathways of m^6^A phenotype-related DEGs, we employed KEGG pathway enrichment analysis (**Figure 6C**).

### m^6^A phenotype-related DEGs in OA

**Figure 7:**
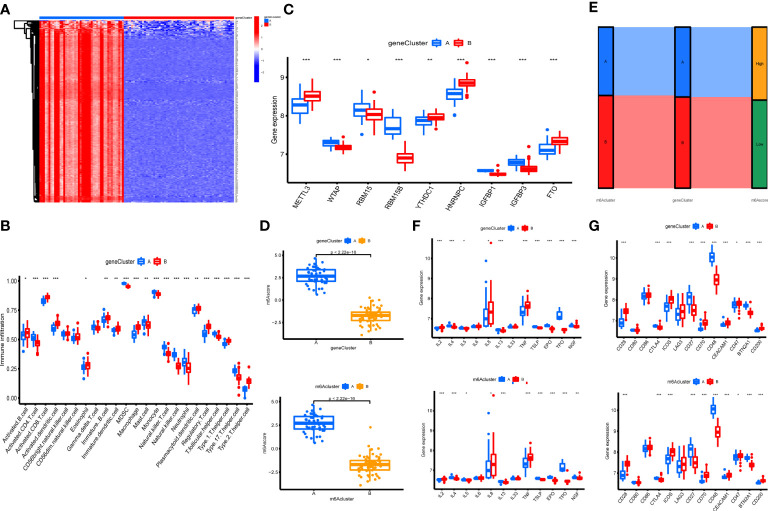
Construction of m^6^A signatures. **(A)** Heatmap of m^6^A phenotype-related in different gene clusters. **(B)** The immunocytes infiltrating levels in distinct gene clusters (*P < 0.05, **P < 0.01, ***P < 0.001). **(C)** The expression levels of 9 key m^6^A regulators in different gene clusters (*P < 0.05, **P < 0.01, ***P < 0.001). **(D)** The m^6^A score in different gene clusters and m^6^A clusters. **(E)** Alluvial diagram showing the changes in m^6^A clusters, gene clusters, and m^6^A score. **(F)** Differences in the expression of immune regulatory genes in different gene clusters and m^6^A clusters (*P < 0.05, **P < 0.01, ***P < 0.001). **(G)** Differences in the expression of immune checkpoints in different gene clusters and m^6^A clusters (*P < 0.05, **P < 0.01, ***P < 0.001).

The 303 m^6^A phenotype-associated DEGs were analyzed by unsupervised clustering analysis. It was most appropriate to divide the sample into two gene clusters (**Figure 2S**). There were considerable discrepancies between the two gene clusters, and only one m^6^A methylation-related gene was highly expressed in cluster B (**Figure 7A**). The evident difference in m^6^A phenotype-related gene expression between the two gene clusters was significant in controlling the establishment of immune cell infiltration (**Figure 7B**). The m^6^A regulators also had significantly different expression in different gene clusters (**Figure 7C**). However, these assays were unable to predict the m^6^A methylation pattern in particular individuals. We developed an m^6^A scoring system to quantify the pattern of m^6^A modifications in each OA patient. m^6^A cluster A had a considerably higher m^6^A score than m^6^A cluster B. Furthermore, the m^6^A score was considerably higher in gene cluster A. The m^6^A score in gene cluster A was significantly higher than that in gene cluster B (**Figure 7D**). An alluvial plot was used to display the characteristic variations in each patient with OA. The results of m^6^A regulators typing were similar to those of genotyping (**Figure 7E**). We also analyzed the differential expression of immune regulatory genes and immune checkpoints in m^6^A clusters and gene clusters. There are significant differences in the expression of immune regulatory genes and immune checkpoints between A and B (**Figures 7F, G**).

### Predictive model in OA

**Figure 8:**
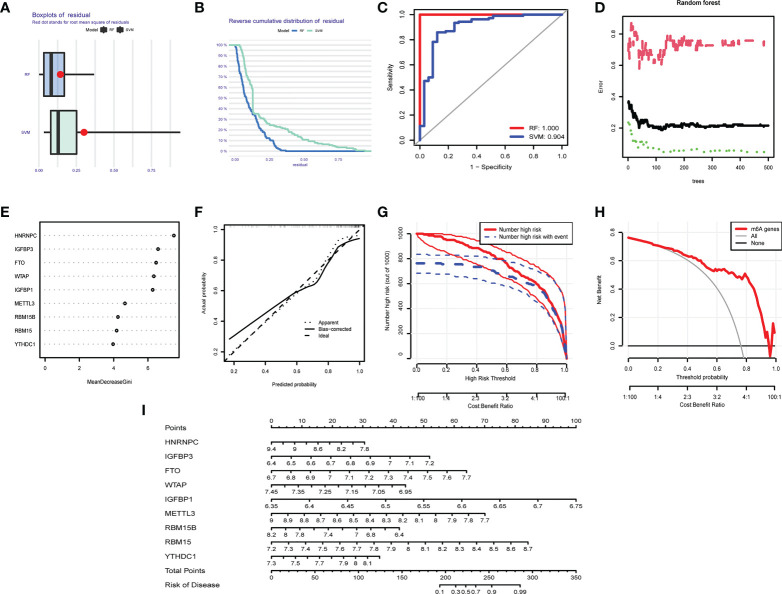
Predictive model in OA. **(A)** Boxplot of RFM and SVMM residuals (the smaller the number, the better the model). **(B)** Reverse cumulative distribution map of RFM and SVMM residuals (The smaller the number is, the better the model is). **(C)** Evaluation of the accuracy of RFM and SVMM by receiver operating characteristic (ROC) diagram (the larger the area is, the higher the accuracy is). **(D)** The number of optimal trees in the verification error analysis of the random forest tree map (selecting the tree value with the lowest error). **(E)** The importance score of m^6^A regulators. **(F)** The correction curve of the prediction model (the closer to the ideal dotted line, the more reliable the result). **(G)** Clinical impact curve to judge the accuracy of the prediction model (red line for the model to predicted high-risk patients, blue dotted line for actual high-risk patients). **(H)** The accuracy of the decision curve detection model (the farther the end point of the red line is from the gray line, the higher the accuracy is). **(I)** OA prediction model based on m^6^A regulators.

We compared the advantages of the random forest model (RFM) and support vector machine model (SVMM). The RFM was better than the SVMM (**Figures 8A–C**). The optimal cutoff point was selected for analysis according to the random forest tree model. The importance score of m^6^A regulators was obtained (**Figures 8D, E**). An OA prediction model was established and evaluated. The calibration curve, clinical influence curve and decision curve all showed that the model was accurate (**Figures 8F–H**). The predictive model can predict the incidence of OA disease by score (**Figure 8I**).

### Expression of m^6^A regulator in two chondrocyte lines after the treatment of IL-1β

**Figure 9:**
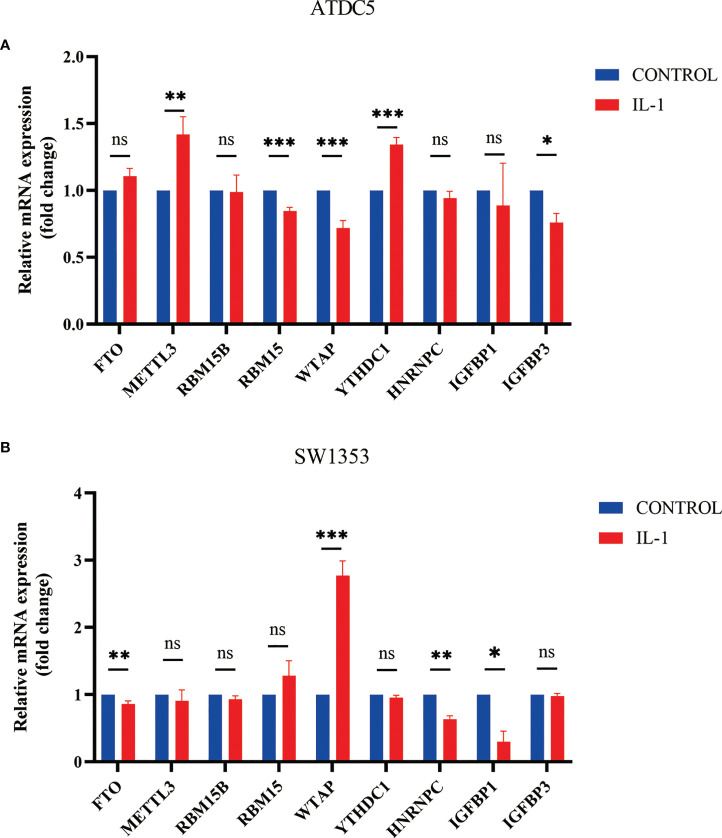
Expression of m^6^A regulator in cell model of OA. **(A)** Column diagram of m^6^A regulator in ATDC5 before and after treatment by IL-1β (*P<0.05, **P<0.01, ***P<0.001). **(B)** Column diagram of m^6^A regulator in SW1353 before and after treatment by IL-1β ns, P<0.05, No statistical difference,*P>0.05, **P>0.01, ***P>0.001.

After we found that m^6^A regulators play an important role in OA, we established an *in vitro* cell model of OA by treating SW1353 cells and ATDC5 cells with IL-1β. The differences in m^6^A regulator gene expression before and after cell treatment were assessed. After treatment of IL-1β, three m^6^A regulators (IGFBP3, RBM15, and WTAP) were down-regulated and two m^6^A regulators (METTL3 and YTHDC1) were up-regulated in ATDC5. In SW1353 cells after treatment with IL-1β, the expression of FTO, HNRNPC, and IGFBP1 was down-regulated, and the expression of WTAP was up-regulated (**Figures 9A, B**). It appeared that the gene expression in the OA model established with cell lines is similar to that of the m^6^A cluster B. These may be related to the accumulation of macrophages in the m^6^A cluster B, and IL-1 is mainly secreted by macrophages.

## Discussion

OA is a prevalent degenerative joint disease that can be painful and uncomfortable (36). Increasing evidence shows that m^6^A modification is critical for inflammation and innate immunity by interacting with a variety of m^6^A regulators. Abnormal modification of the m^6^A gene may lead to disorders of important genes and dynamic balance, resulting in disease (37). In recent years, there have been many discussions about the relationship between inflammatory factors and the pathogenesis and progression of OA. OA is now regarded as an inflammatory illness defined by inflammatory factors rather than a degenerative disease (38). The inflammatory environment is critical to the progression of OA (39). At present, researchers are interested in the association between m^6^A modifications and OA. Thus, future studies will focus on the link between epigenetic regulation and inflammatory factors in OA.

We systematically investigated the mechanism of the modification mode of m^6^A in the immune microenvironment of OA. First, we discovered that the expression of the majority of m^6^A regulators changed between healthy controls and OA patients, indicating that m^6^A regulators were implicated in the occurrence and progression of OA. Previous studies have indicated that METTL3 plays a significant role in the pathogenesis of osteoarthritis. Liu’s study demonstrated that METTL3 regulates OA pathogenesis by promoting inflammation and extracellular matrix (ECM) synthesis (7). Jiangdong Ren showed that METTL3-mediated LINC00680 accelerates OA (40). However, Sang. W showed that overexpression of METTL3 leads to reduced inflammatory cytokine levels and regulates the TIMP/MMP balance (14). There are a few studies on the relationship between FTO and OA. However, there is also controversy between these studies (41, 42). In our study, both FTO and METTL3 were found to be key m^6^A regulators in OA. Our study shows that METTL3 is downregulated and FTO is upregulated in OA.

We further classified OA patients according to the screening results for 9 key m^6^A regulators. Furthermore, several m^6^A regulators exhibited expression correlations with each other, which revealed the m^6^A modification regulatory network. Third, the impact of m^6^A modification patterns on immunocytes infiltration was determined to strengthen the knowledge of the interaction between m^6^A RNA and the immunological response. We found that IGFBP1 and RBM15B were strongly correlated with infiltrating immunocytes in OA. Lange-Brokaar’s study showed that the immunocytes participating in cartilage injury and repair mostly comprise T cells, B cells, NK cells, DCs and macrophages (43). Our study also showed that these immunocytes play a significant role in distinct m^6^A modification patterns. The m^6^A cluster B had more kinds of infiltrating immunocytes than cluster A, and the degree of immune cell infiltration could not be judged. However, these validated the accuracy of our immunophenotypic categorization through key m^6^A regulators. It also showed that there is a certain correlation between the m^6^A regulator and immune infiltration. Different immune microenvironments has different effects on osteoarthritis, which also provides a basis for immune research in osteoarthritis.

Furthermore, the m^6^A phenotype-related DEGs and their biological functions were identified. Our study confirmed that m^6^A phenotype-related DEGs were significantly related to immune cell infiltration. We further found that the different expression levels of immune regulatory and immune checkpoints in different m^6^A clusters and gene clusters. This finding may provide new ideas for the study on treatment of OA. We set up an m^6^A score model to assess m^6^A modifications in individual OA patients. We also found that the m^6^A clusters and gene clusters contained almost the same sets of patients. Then, we established a prediction model that can predict the occurrence and progression of OA. We can score patients according to the expression of the m^6^A regulator, and then get the probability of OA by score. Finally, we tried to use the OA cell model to verify whether the changes in the m^6^A gene were consistent with our bioinformatics analysis. The results of real-time PCR analysis were not consistent with the differences in m^6^A regulator expression between OA patients and healthy patients. This may be due to the fact that the cell model was different from the human body and that the samples came from the peripheral blood of the patient. But the change of m^6^A regulators’ expression proved that these genes play a role in the progression of OA. More importantly, we found the change in gene expression in the OA cell model was almost consistent with that of the m^6^A cluster B. We believed that this phenomenon is due to the fact that we used IL-1β for cell modeling, and the macrophages that mainly secrete IL-1 were mainly enriched in the B cluster.

The study of m^6^A regulators is widely used in the field of oncology, and better treatment can be achieved through molecular subtyping (44, 45). In the field of OA, however, few studies have focused on the mechanism of m^6^A regulators. We are the first to systematically analyze the mechanism of m^6^A in OA. We proved that m^6^A modification has a role in regulating the OA immune microenvironment.

### Limitations of the study

These results will provide new inspiration for the study of the pathogenesis and treatment of OA. However, there are still some deficiencies in our research. First, our study is based on bioinformatics analysis, and although our results are accurate and reliable in theory, they need to be verified by more experiments. In the future, we hope that single-cell RNA seq can be performed to better understand how m^6^A modification affects immune cell infiltration in OA patients. In addition, the expression profile dataset (GSE48556) contains only 139 samples, and a larger sample size would be more beneficial for bioinformatics analysis. Therefore, we hope that more OA expression profile data and studies with larger sample sizes will become available. Third, because OA is a nonneoplastic disease, its survival curve is rarely studied, so it is impossible to establish a model considering the m^6^A score and survival. However, the severity of knee joint damage can be studied, and more clinical data will establish the relationship between the m^6^A score and disease progression. In this way, m^6^A score will be critical in predicting the progression of the disease. But the accuracy of m^6^A scoring model need validated by a large number of patient samples. Finally, there were few genes in the GO and KEGG analyses of m^6^A phenotype-related DEGs, which could be due to the limited sample size. However, some of the enriched biological functions mentioned in our study have been proven to participate in OA by other studies, which indicates that our results are worth using for reference. We hope that the expression profile datasets with a larger sample size become available.

## Conclusion

In summary, our study reveals for the first time the potential regulatory mechanism of m^6^A methylation modification in the immune microenvironment of OA. This study also sheds new light on the pathogenesis of OA. The difference in m^6^A modification mode is a significant contributor to the complexity of the OA microenvironment. Individual OA patients can utilize the m^6^A score to assess their m^6^A modification pattern and immune cell infiltration features. The prediction model we have established is helpful in predicting disease development in OA patients. Additional studies are needed to fully clarify the molecular mechanisms of m^6^A regulation and biological function during OA.

## Data availability statement

Publicly available datasets were analyzed in this study. This data can be found here: The GSE48556 datasets were obtained from the GEOdatabase(https://www.ncbi.nlm.nih.gov/geo/query/acc.cgi?acc=GSE48556).

## Author contributions

GS and YO designed this study. TW, HF and SC conducted literature searches and management. YO, YT and HM were responsible for data management and statistical analysis. GZ, ZW, WY interpreted the findings. YO performed the manuscript writing. The manuscript was revised by all authors, and the final version was approved by all. All authors contributed to the article and approved the submitted version.

## Funding

This work was supported by the National Natural Science Foundation of China (NO.81960881), which was hosted by GS. The funders were not involved in the study design, data collection, statistical analysis or writing of the manuscript. The GSE48556 datasets were obtained from the GEO database (https://www.ncbi.nlm.nih.gov/geo/query/acc.cgi?acc=GSE48556).

## Conflict of interest

The authors declare that the research was conducted in the absence of any commercial or financial relationships that could be construed as a potential conflict of interest.

## Publisher’s note

All claims expressed in this article are solely those of the authors and do not necessarily represent those of their affiliated organizations, or those of the publisher, the editors and the reviewers. Any product that may be evaluated in this article, or claim that may be made by its manufacturer, is not guaranteed or endorsed by the publisher.

## Supplementary material

Figure 1S The best m6A cluster number was evaluated by the CCM. **(A–G)** K=3-9 CCM.

Figure 2S The best gene cluster number was evaluated by the CCM. **(A–I)** K=2-9 CCM. **(J)** Relative change in the area under the CDF curve for k from 2 to 9.

## References

[1] Glyn-Jones S Palmer AJ Agricola R Price AJ Vincent TL Weinans H. Osteoarthritis. Lancet (2015) 386:376–87. doi: 10.1016/S0140-6736(14)60802-3 25748615

[2] Nelson AE. Osteoarthritis year in review 2017: clinical. Osteoarthritis Cartilage (2018) 26:319–25. doi: 10.1016/j.joca.2017.11.014 PMC5835411 29229563

[3] Sharma L. Osteoarthritis of the knee. N Engl J Med (2021) 384:51–9. doi: 10.1056/NEJMcp1903768 33406330

[4] Hunter DJ Schofield D Callander E. The individual and socioeconomic impact of osteoarthritis. Nat Rev Rheumatol (2014) 10:437–41. doi: 10.1038/nrrheum.2014.44 24662640

[5] Li KC Hu YC. Cartilage tissue engineering: recent advances and perspectives from gene regulation/therapy. Adv Healthc Mater (2015) 4:948–68. doi: 10.1002/adhm.201400773 25656682

[6] Woodell-May JE Sommerfeld SD. Role of inflammation and the immune system in the progression of osteoarthritis. J Orthop Res (2020) 38:253–7. doi: 10.1002/jor.24457 31469192

[7] Liu Q Li M Jiang L Jiang R Fu B. METTL3 promotes experimental osteoarthritis development by regulating inflammatory response and apoptosis in chondrocyte. Biochem Biophys Res Commun (2019) 516:22–7. doi: 10.1016/j.bbrc.2019.05.168 31186141

[8] Harvey ZH Chen Y Jarosz DF. Protein-based inheritance: Epigenetics beyond the chromosome. Mol Cell (2018) 69:195–202. doi: 10.1016/j.molcel.2017.10.030 29153393 PMC5775936

[9] Teng PC Liang Y Yarmishyn AA Hsiao YJ Lin TY Lin TW. RNA Modifications and epigenetics in modulation of lung cancer and pulmonary diseases. Int J Mol Sci (2021) 22:(19):10592. doi: 10.3390/ijms221910592 34638933 PMC8508636

[10] Zhang W He L Liu Z Ren X Qi L Wan L. Multifaceted functions and novel insight into the regulatory role of RNA N(6)-methyladenosine modification in musculoskeletal disorders. Front Cell Dev Biol (2020) 8:870. doi: 10.3389/fcell.2020.00870 32984346 PMC7493464

[11] Zhang X Zhang S Yan X Shan Y Liu L Zhou J. m6A regulator-mediated RNA methylation modification patterns are involved in immune microenvironment regulation of periodontitis. J Cell Mol Med (2021) 25:3634–45. doi: 10.1111/jcmm.16469 PMC8034465 33724691

[12] Huang X Lv D Yang X Li M Zhang H. m6A RNA methylation regulators could contribute to the occurrence of chronic obstructive pulmonary disease. J Cell Mol Med (2020) 24:12706–15. doi: 10.1111/jcmm.15848 PMC7686997 32961012

[13] Li H Xiao W He Y Wen Z Cheng S Zhang Y. Novel insights into the multifaceted functions of RNA n(6)-methyladenosine modification in degenerative musculoskeletal diseases. Front Cell Dev Biol (2021) 9:766020. doi: 10.3389/fcell.2021.766020 35024366 PMC8743268

[14] Sang W Xue S Jiang Y Lu H Zhu L Wang C. METTL3 involves the progression of osteoarthritis probably by affecting ECM degradation and regulating the inflammatory response. Life Sci (2021) 278:119528. doi: 10.1016/j.lfs.2021.119528 33894271

[15] Yi L Wu G Guo L Zou X Huang P. Comprehensive analysis of the PD-L1 and immune infiltrates of m(6)A RNA methylation regulators in head and neck squamous cell carcinoma. Mol Ther Nucleic Acids (2020) 21:299–314. doi: 10.1016/j.omtn.2020.06.001 32622331 PMC7332506

[16] Yang Y Hsu PJ Chen YS Yang YG. Dynamic transcriptomic m(6)A decoration: writers, erasers, readers and functions in RNA metabolism. Cell Res (2018) 28:616–24. doi: 10.1038/s41422-018-0040-8 PMC5993786 29789545

[17] Shulman Z Stern-Ginossar N. The RNA modification N(6)-methyladenosine as a novel regulator of the immune system. Nat Immunol (2020) 21:501–12. doi: 10.1038/s41590-020-0650-4 32284591

[18] Liu ZX Li LM Sun HL Liu SM. Link between m6A modification and cancers. Front Bioeng Biotechnol (2018) 6:89. doi: 10.3389/fbioe.2018.00089 30062093 PMC6055048

[19] Ma X Li Y Wen J Zhao Y. m6A RNA methylation regulators contribute to malignant development and have a clinical prognostic effect on cervical cancer. Am J Transl Res (2020) 12:8137–46. PMC7791487 33437387

[20] Shen S Zhang R Jiang Y Li Y Lin L Liu Z. Comprehensive analyses of m6A regulators and interactive coding and non-coding RNAs across 32 cancer types. Mol Cancer (2021) 20:67. doi: 10.1186/s12943-021-01362-2 33849552 PMC8045265

[21] Wang J Gao F Zhao X Cai Y Jin H. Integrated analysis of the transcriptome-wide m6A methylome in preeclampsia and healthy control placentas. PeerJ (2020) 8:e9880. doi: 10.7717/peerj.9880 32983644 PMC7500358

[22] Sun D Yang H Fan L Shen F Wang Z. m6A regulator-mediated RNA methylation modification patterns and immune microenvironment infiltration characterization in severe asthma. J Cell Mol Med (2021) 25:10236–47. doi: 10.1111/jcmm.16961 PMC8572790 34647423

[23] Wang J Wang K Liu W Cai Y Jin H. m6A mRNA methylation regulates the development of gestational diabetes mellitus in han Chinese women. Genomics (2021) 113:1048–56. doi: 10.1016/j.ygeno.2021.02.016 33667648

[24] Han D Liu J Chen C Dong L Liu Y Chang R. Anti-tumour immunity controlled through mRNA m(6)A methylation and YTHDF1 in dendritic cells. Nature (2019) 566:270–4. doi: 10.1038/s41586-019-0916-x PMC6522227 30728504

[25] Wang L Wen M Cao X. Nuclear hnRNPA2B1 initiates and amplifies the innate immune response to DNA viruses. Science (2019) 365(6454):eaav0758. doi: 10.1126/science.aav0758 31320558

[26] Lin S Xu H Zhang A Ni Y Xu Y Meng T. Prognosis analysis and validation of m(6)A signature and tumor immune microenvironment in glioma. Front Oncol (2020) 10:541401. doi: 10.3389/fonc.2020.541401 33123464 PMC7571468

[27] Jin Y Wang Z He D Zhu Y Hu X Gong L. Analysis of m6A-related signatures in the tumor immune microenvironment and identification of clinical prognostic regulators in adrenocortical carcinoma. Front Immunol (2021) 12:637933. doi: 10.3389/fimmu.2021.637933 33746977 PMC7966528

[28] Li M Yin H Yan Z Li H Wu J Wang Y. The immune microenvironment in cartilage injury and repair. Acta Biomater (2022) 140:23–42. doi: 10.1016/j.actbio.2021.12.006 34896634

[29] Ramos YF Bos SD Lakenberg N Böhringer S Den Hollander WJ Kloppenburg M. Genes expressed in blood link osteoarthritis with apoptotic pathways. Ann Rheum Dis (2014) 73:1844–53. doi: 10.1136/annrheumdis-2013-203405 23864235

[30] Wu H Dong H Fu Y Tang Y Dai M Chen Y. Expressions of m6A RNA methylation regulators and their clinical predictive value in cervical squamous cell carcinoma and endometrial adenocarcinoma. Clin Exp Pharmacol Physiol (2021) 48:270–8. doi: 10.1111/1440-1681.13412 33006785

[31] Xiong W Li C Wan B Zheng Z Zhang Y Wang S. N6-methyladenosine regulator-mediated immue patterns and tumor microenvironment infiltration characterization in glioblastoma. Front Immunol (2022) 13:819080. doi: 10.3389/fimmu.2022.819080 35359993 PMC8961865

[32] Charoentong P Finotello F Angelova M Mayer C Efremova M Rieder D. Pan-cancer immunogenomic analyses reveal genotype-immunophenotype relationships and predictors of response to checkpoint blockade. Cell Rep (2017) 18:248–62. doi: 10.1016/j.celrep.2016.12.019 28052254

[33] Sotiriou C Wirapati P Loi S Harris A Fox S Smeds J. Gene expression profiling in breast cancer: understanding the molecular basis of histologic grade to improve prognosis. J Natl Cancer Inst (2006) 98:262–72. doi: 10.1093/jnci/djj052 16478745

[34] Zeng D Li M Zhou R Zhang J Sun H Shi M. Tumor microenvironment characterization in gastric cancer identifies prognostic and immunotherapeutically relevant gene signatures. Cancer Immunol Res (2019) 7:737–50. doi: 10.1158/2326-6066.CIR-18-0436 30842092

[35] Legendre F Baugé C Roche R Saurel AS Pujol JP. Chondroitin sulfate modulation of matrix and inflammatory gene expression in IL-1beta-stimulated chondrocytes–study in hypoxic alginate bead cultures. Osteoarthritis Cartilage (2008) 16:105–14. doi: 10.1016/j.joca.2007.05.020 17625924

[36] Bloomfield RA Fennema MC Mcisaac KA Teeter MG. Proposal and validation of a knee measurement system for patients with osteoarthritis. IEEE Trans BioMed Eng (2019) 66:319–26. doi: 10.1109/TBME.2018.2837620 29993529

[37] Liu Y Guo X Zhao M Ao H Leng X Liu M. Contributions and prognostic values of m(6) a RNA methylation regulators in non-small-cell lung cancer. J Cell Physiol (2020) 235:6043–57. doi: 10.1002/jcp.29531 32052446

[38] Jiang Y. Osteoarthritis year in review 2021: biology. Osteoarthritis Cartilage (2022) 30:207–15. doi: 10.1016/j.joca.2021.11.009 34801671

[39] Wang P Meng Q Wang W Zhang S Xiong X Qin S. Icariin inhibits the inflammation through down-regulating NF-κB/HIF-2α signal pathways in chondrocytes. Biosci Rep (2020) 40(11):BSR20203107. doi: 10.1042/BSR20203107 33155655 PMC7685011

[40] Ren J Li Y Wuermanbieke S Hu S Huang G. N(6)-methyladenosine (m(6)A) methyltransferase METTL3-mediated LINC00680 accelerates osteoarthritis through m(6)A/SIRT1 manner. Cell Death Discovery (2022) 8:240. doi: 10.1038/s41420-022-00890-0 35501316 PMC9061755

[41] Panoutsopoulou K Metrustry S Doherty SA Laslett LL Maciewicz RA Hart DJ. The effect of FTO variation on increased osteoarthritis risk is mediated through body mass index: a mendelian randomisation study. Ann Rheum Dis (2014) 73:2082–6. doi: 10.1136/annrheumdis-2013-203772 PMC4251538 23921993

[42] Dai J Ying P Shi D Hou H Sun Y Xu Z. FTO variant is not associated with osteoarthritis in the Chinese han population: replication study for a genome-wide association study identified risk loci. J Orthop Surg Res (2018) 13:65. doi: 10.1186/s13018-018-0769-2 29606151 PMC5879643

[43] De Lange-Brokaar BJ Ioan-Facsinay A Van Osch GJ Zuurmond AM Schoones J Toes RE. Synovial inflammation, immune cells and their cytokines in osteoarthritis: a review. Osteoarthritis Cartilage (2012) 20:1484–99. doi: 10.1016/j.joca.2012.08.027 22960092

[44] Li B Cui Y Nambiar DK Sunwoo JB Li R. The immune subtypes and landscape of squamous cell carcinoma. Clin Cancer Res (2019) 25:3528–37. doi: 10.1158/1078-0432.CCR-18-4085 PMC6571041 30833271

[45] Zhang B Wu Q Li B Wang D Wang L Zhou YL. m(6)A regulator-mediated methylation modification patterns and tumor microenvironment infiltration characterization in gastric cancer. Mol Cancer (2020) 19:53. doi: 10.1186/s12943-020-01170-0 32164750 PMC7066851

