# Supplementary figures and images for: Characterization of immune microenvironment infiltration and m6A regulator-mediated RNA methylation modification patterns in osteoarthritis
Source: Front Immunol. 2022 Nov 23;13:1018701. doi: 10.3389/fimmu.2022.1018701 (PMC9728527; doi:10.3389/fimmu.2022.1018701)

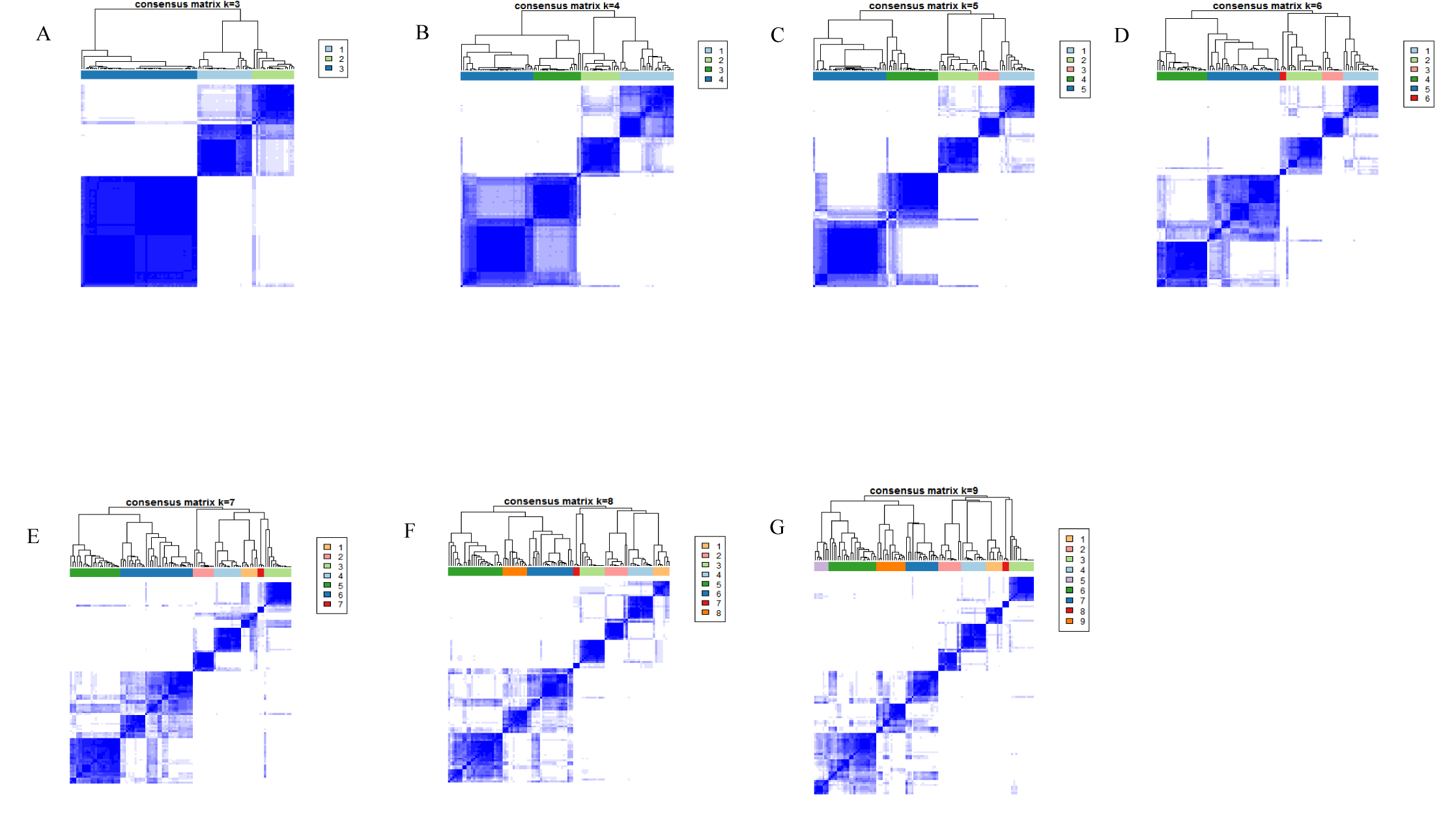

Supplement: Figure 1S — The best m6A cluster number was evaluated by the CCM. (A–G) K=3-9 CCM. [file Image_1.tif]

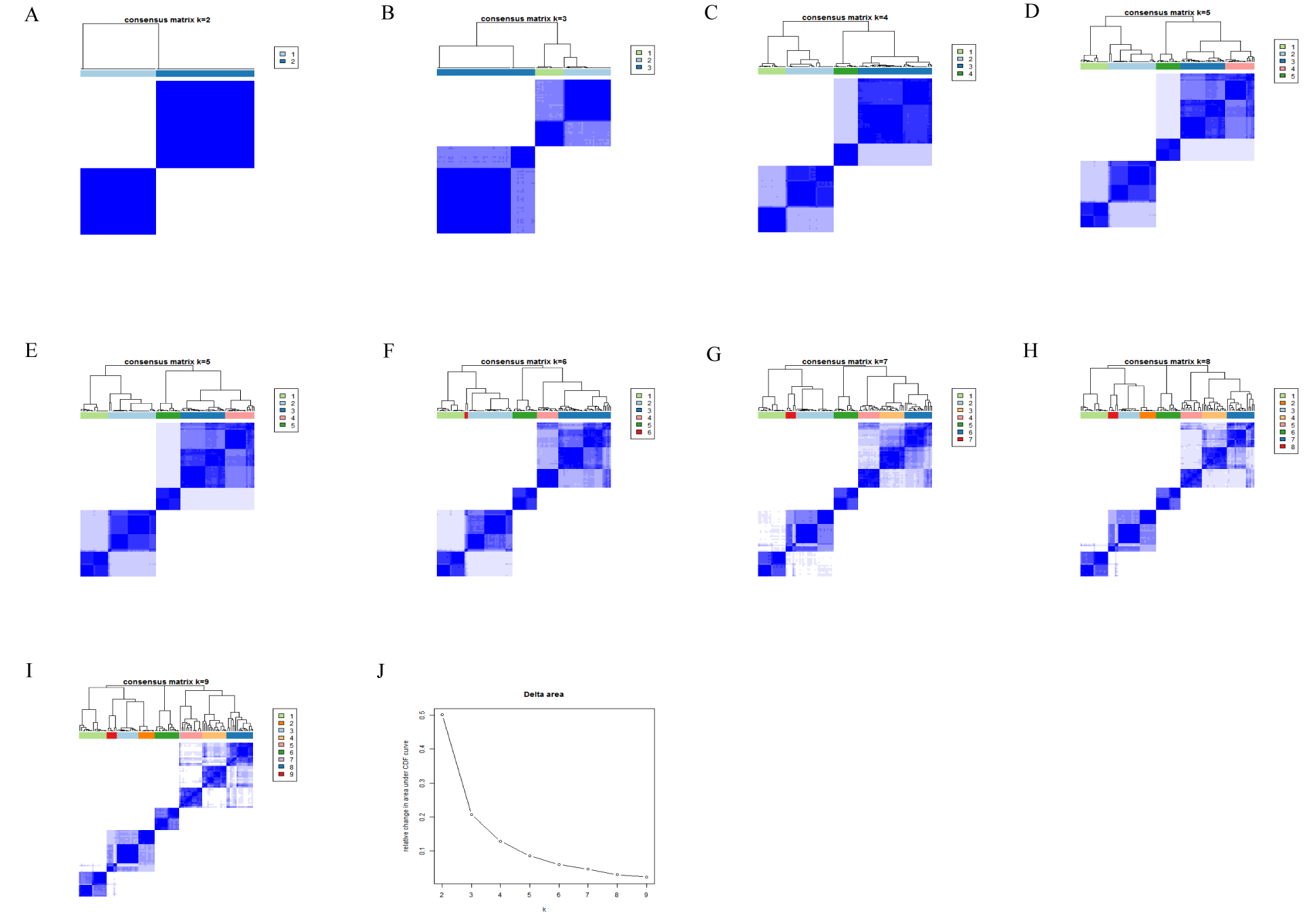

Supplement: Figure 2S — The best gene cluster number was evaluated by the CCM. (A–I) K=2-9 CCM. (J) Relative change in the area under the CDF curve for k from 2 to 9. [file Image_2.tif]
